# Grating-based x-ray dark-field CT for lung cancer diagnosis in mice

**DOI:** 10.1186/s41747-023-00399-w

**Published:** 2024-01-25

**Authors:** Peiyuan Guo, Li Zhang, Jincheng Lu, Huitao Zhang, Xiaohua Zhu, Chengpeng Wu, Xiangwen Zhan, Hongxia Yin, Zhenchang Wang, Yan Xu, Zhentian Wang

**Affiliations:** 1 Department of Engineering Physics, Tsinghua University, Beijing, China; 2 Key Laboratory of Particle & Radiation Imaging (Tsinghua University) of Ministry of Education, Beijing, China; 3 Institute for Precision Medicine, Tsinghua University, Beijing, China; 4 School of Mathematical Sciences, Capital Normal University, Beijing, China; 5 NHC Key Laboratory of Human Disease Comparative Medicine, Beijing Engineering Research Center for Experimental Animal Models of Human Critical Diseases, Institute of Laboratory Animal Sciences, Chinese Academy of Medical Sciences (CAMS) and Comparative Medicine Center, Peking Union Medical College (PUMC), Beijing, China; 6 Department of Radiology, Beijing Friendship Hospital, Capital Medical University, Beijing, China

**Keywords:** Disease progression, Early detection of cancer, Lung diseases, Tomography (x-ray computed), X-rays

## Abstract

**Background:**

The low absorption of x-rays in lung tissue and the poor resolution of conventional computed tomography (CT) limits its use to detect lung disease. However, x-ray dark-field imaging can sense the scattered x-rays deflected by the structures being imaged. This technique can facilitate the detection of small alveolar lesions that would be difficult to detect with conventional CT. Therefore, it may provide an alternative imaging modality to diagnose lung disease at an early stage.

**Methods:**

Eight mice were inoculated with lung cancers simultaneously. Each time two mice were scanned using a grating-based dark-field CT on days 4, 8, 12, and 16 after the introduction of the cancer cells. The detectability index was calculated between nodules and healthy parenchyma for both attenuation and dark-field modalities. High-resolution micro-CT and pathological examinations were used to crosscheck and validate our results. Paired *t*-test was used for comparing the ability of dark-field and attenuation modalities in pulmonary nodule detection.

**Results:**

The nodules were shown as a signal decrease in the dark-field modality and a signal increase in the attenuation modality. The number of nodules increased from day 8 to day 16, indicating disease progression. The detectability indices of dark-field modality were higher than those of attenuation modality (*p* = 0.025).

**Conclusions:**

Compared with the standard attenuation CT, the dark-field CT improved the detection of lung nodules.

**Relevance statement:**

Dark-field CT has a higher detectability index than conventional attenuation CT in lung nodule detection. This technique could improve the early diagnosis of lung cancer.

**Key points:**

• Lung cancer progression was observed using x-ray dark-field CT.

• Dark-field modality complements with attenuation modality in lung nodule detection.

• Dark-field modality showed a detectability index higher than that attenuation in nodule detection.

**Graphical Abstract:**

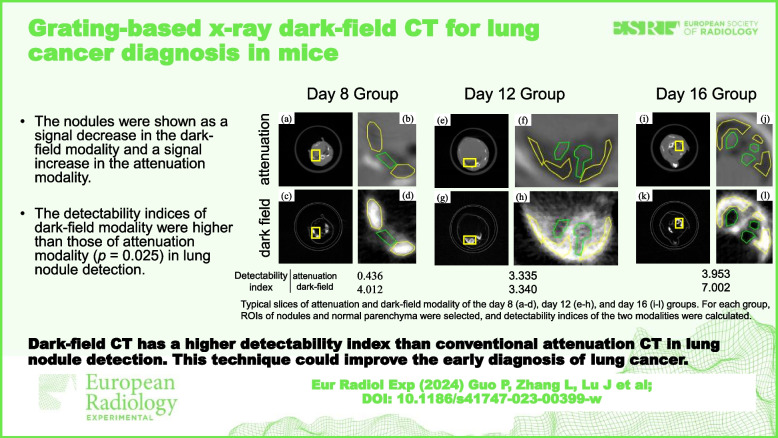

## Background

Lung diseases such as chronic obstructive pulmonary disease and lung cancer are common causes of death worldwide [1–5]. X-ray radiography and computed tomography (CT) are noninvasive tools to diagnose lung disease. In radiography, images are generated based on the differential attenuation of x-rays with different tissue densities. However, due to the limited spatial resolution of the CT and the poor attenuation of x-ray in lung tissue, the standard CT technique cannot always detect small structural changes in the pulmonary alveoli [4, 6] and very small lung tumors [7, 8]. Therefore, early diagnosis of lung diseases like emphysema can only be done by observing the slight signal change of attenuation caused by structural change of alveoli parenchyma [9].

Grating-based x-ray dark-field imaging utilizes x-ray’s wave character [10–13] and detects the perturbation of the wavefront of x-rays. It measures the phase contrast signal (representing refraction) and dark-field signal (representing small angle scattering) caused by an object [14]. Although lung tissue tends to have low x-ray attenuation, the alveoli have strong scattering properties [15, 16]. As a result, grating-based x-ray dark-field imaging can identify pathological changes within the lung that generate a strong dark-field signal [15–18] and could be used to identify lung diseases at an early stage.

Dark-field imaging has already been experimentally used to assess bones, brain, microcalcifications, and lungs in humans [17, 19–23]. Scherer et al. [24] compared *in vivo* dark-field radiography with standard attenuation radiography in a lung cancer mouse model and found that x-ray dark-field imaging is far more suitable for nodule detection. Other studies have also found that dark-field imaging can improve the early detection of emphysema and fibrosis [6]. Furthermore, pathological changes in human lung could be differentiated with spectral dark-field radiography [25]. Velroyen et al. [26] conducted the first dark-field CT on mice and demonstrated that emphysema and fibrosis can be detected and classified with the cooperation of dark-field and attenuation modality. Furthermore, *in vivo* dark-field CT was also conducted to detect lung diseases in mice [27]. Apart from lung disease research on the scale of mice, a prototype of dark-field CT with a scale of humans has been recently developed [21], which carried forward the application of clinical diagnosis of lung diseases.

In this study, we aimed to evaluate the ability of the dark-field CT to detect pulmonary nodules in a mouse lung cancer model at different stages of cancer development in relation to standard attenuation modality and micro-CT.

## Methods

### Preparation of the mouse models

This prospective study was approved by our institutional review board. All experiments were performed as per GB/T 35892-2018 Laboratory Animal-Guideline for ethical review of animal welfare.

For this study, 5-week-old male BALB/c (Hfk, Beijing, China) nude mice were used. The mice were housed in specific pathogen free rooms at a constant temperature of 20 to 26 °C and humidity of 40% to 70% with a 12-h light cycle. The mice were provided with food and water ad libitum. Lung cancer was introduced into the mice by injecting a 100 μL (0.5 × 10^6^ cells) solution of the B16 cell line (Institute of Laboratory Animals Science, Peking Union Medical College, Beijing, China) into the caudal vein [28]. The eight mice were injected simultaneously, and to eliminate the influence of inherent variations among different mice, each time two mice were randomly selected and scanned using a dark-field CT on days 4, 8, 12, and 16 after the introduction of the cancer cells.

For the autolysis study (details are given in dedicated subsections below), another four mice were used. The origin of these mice was the same as those mentioned above, except that they were not injected with cancer cells.

### Grating-based dark-field CT

The dark-field signal is related to the strength of small-angle scattering. For porous objects, dark-field signal is generated by the microstructure containing the mass interfaces between the object and the air [29]. When exposed to x-rays, the lungs can cause a strong small-angle scattering at the pulmonary alveoli structure on the micron scale, thus creating a strong dark-field signal. In lung cancer, the porous alveoli structure is replaced by relatively denser and less scattering nodules, eventually leading to a decrease in the dark-field signal and an increase in the attenuation signal [24].

**Fig. 1:**
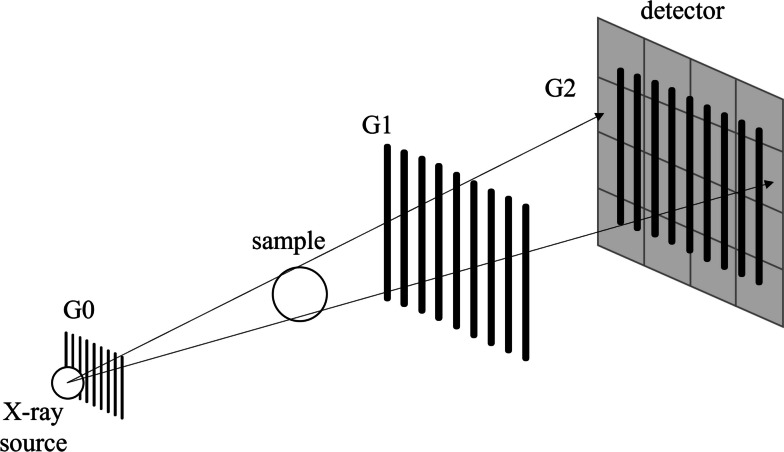
Schematic diagram illustrating the positions of the three gratings of the interferometer. The source grating (G0), the beam splitter (G1), and the analyzer grating (G2) are placed in the beam path to modulate the wavefront

A standard grating interferometer setup with three gratings can be used to measure the dark-field signal, as shown in Fig. 1. The core of these three gratings is the beam splitter grating G1, which generates a periodic intensity fringe pattern at a certain distance behind G1, called the Talbot effect [13, 30]. Since the period of the fringes is much smaller than the pixel of the detector, the analyzer grating G2 is used to sample the fringe pattern by blocking part of the incoming x-rays. Since this method requires high coherence of the x-ray source, a source grating G0 can be placed right after the x-ray source to generate an array of line sources, each of which is partially coherent, and this G0 helps laboratory source to satisfy the coherence requirement [31]. The G2 grating moves step by step perpendicular to the fringe to absorb different parts of the fringe pattern, and the detector records the intensity at each step, forming an intensity curve relative to the steps called the phase stepping curve [14]. Fourier analysis is then applied to the phase stepping curve to calculate the fringe’s mean intensity, phase, and visibility, which correspond to the three modalities of this imaging method.

If a sample is placed in the beam path, it will cause attenuation of the beam, leading to a reduction in the intensity, which is used in conventional imaging. Additionally, the small-angle scattering can be caused by the internal microstructures within the sample, leading to a reduction in the visibility of the fringes. The dark-field signal of a sample is defined as the visibility reduction and is calculated by taking the negative logarithm of the ratio between the visibility of the fringes with and without the sample.

As for the image reconstruction of dark-field signals, it has been shown that the dark-field signal follows the integral path rule of the exponential decay similar to the Beer-Lambert law [29, 32–34], which is also used on the standard attenuation CT. Therefore, by assuming that the dark-field signal is a rotational-invariant, the reconstruction methods used on the standard CT could also be used for the dark-field reconstruction. However, it is important to note that since the rotational invariance is not seen in many fibrous samples [35–37], this method can lead to artifacts.

### Experimental setup

The experiments were conducted on the experimental setup developed by Tsinghua University. The setup consists of a flat panel detector with a pixel size of 75 μm (DEXELA 2923, Varex Imaging Corporation, Salt Lake City, USA), an x-ray tube with a focal spot of 1.0 mm (MXR-160HP/11, Comet Group, Flamatt, Switzerland), and a set of Talbot-Lau grating interferometer [30]. The three gratings of the interferometer share the same silicon substrate material and the same 0.5-duty cycle. The bar material of G0 and G2 grating was gold, and that of the G1 grating was silicon. The heights of the bars were 47 μm, 38.4 μm, and 40 μm for the G0, G1, and G2, respectively. All gratings had a period of 4.8 μm. The G1 grating produces a phase shift of $\pi$. The distance between the adjacent gratings was set to approximately 0.695 m, using the fifth Talbot order.

### Data acquisition

The mice were euthanized right before the experiment by injecting an overdose of ketamine (Beikang, Jiangxi, China) into the peritoneum. The mice were then fixed in a plastic centrifuge tube. Subsequently, the tube was placed on the rotation stage in an upright position. All images were acquired using a source voltage of 35 kVp and a current of 20 mA. The exposure time of the detector was set at 2,000 ms. Scanning was performed with a magnification factor of about × 1.35. For each scan, 720 angular views were taken at an interval of 0.5°, covering the whole 360°. An 8-step phase stepping was conducted at each angular position. Due to the slow readouts of the detector and the overheads of mechanical moves of motors, the total scan time lasted approximately 8 h. Two set of background phase-stepping images were acquired before and after the rotation. These signals were then used to retrieve the signals from the former and latter half of the 720 angular positions.

### Data processing and reconstruction

The three signals were retrieved by discrete Fourier transform, conducted on each set of phase-stepping images [14, 38]. After retrieval, fan-beam reconstruction can be applied to these three signals to obtain the tomographic images. Due to the tilt of the rotation axis, geometric calibration was necessary before performing the fan beam reconstruction. The exact tilt angle and position of the rotation axis were found through iterative optimization. The three signals were calibrated into a standard fan beam projection according to the position parameters of the rotation axis. Thereafter filtered back projection with a Ram-Lak filter [39] was applied to reconstruct the attenuation and dark-field tomographic images. The CONRAD software [40] was used to reconstruct the projected data into 730 tomographic images with an 850 × 850 matrix, whose isotropic voxels’ edge length is 70 μm.

### Micro-CT

The grating interferometer used in our study had a limited spatial resolution. In order to better visualize the lung cancers in the mice and cross-validate the results of the dark-field CT, micro-CT scans were performed on one of the mice in each group. The micro-CT was conducted on the HR-225CT (Capital Normal University, Beijing, China) using a voltage of 80 kVp and a current of 250 μA. The focal size of the x-ray source in the setup was 8 μm, and the pixel size of the planar detector was 139 μm. The scanning was performed with a magnification factor of about × 13. The projection data of 1,440 angular positions were reconstructed using the Feldkamp-Davis-Kress method [41] into 1,400 tomographic images with a 1,200 × 1,200 matrix, whose isotropic voxels’ edge length was 15 μm. The total acquisition time lasted approximately 26 min.

### Quantitative analysis

We used the detectability index ($d^\prime$) [42–44] to quantitatively evaluate the feasibility of the modalities to detect lung nodule from normal parenchyma. The detectability index is a metric for classification tasks that measures the overlap of the distribution of the two classes on the decision axis, and for an ideal observer, it can be expressed as [42]:

1 $$\begin{array}{c}{d}^{{\prime}2}={K}^{2}\int \frac{{\left|F\left(v\right)\right|}^{2}M{\left(v\right)}^{2}}{N\left(v\right)}dv\end{array}$$

where $K$ is the large-area transfer ratio, $G(v)$ is the spatial frequency spectrum of the difference between the signal and background, $M(v)$ is the system modulation transfer function, and $N(v)$ is the noise power spectrum. However, since the modulation transfer function and noise power spectrum of our system are difficult to measure, we here use another expression to calculate $d^\prime$ [44]:

2 $$\begin{array}{c}{d}^{\prime}=\frac{{\langle S\rangle }_{2}-{\langle S\rangle }_{1}}{\sqrt{\frac{1}{2}{\sigma }_{1}^{2}+\frac{1}{2}{\sigma }_{2}^{2}}}\end{array}$$

where ${\langle S\rangle }_{1}$ and ${\sigma }_{1}$ denotes the mean value and the standard deviation of class 1 and the same for subscript 2 and class 2. Specifically in our study, the class 1 and 2 represent the normal pulmonary parenchyma and nodules, respectively.

In order to measure the $d^\prime$, regions of interest (ROI) have to be selected on CT slices for these two classes. The ROIs are supposed to contain all of the nodule and parenchyma in order to consider the biological variability. However, due to the lack of histological analysis, some areas on the CT slice cannot be definitely classified as nodule or parenchyma, so these areas were excluded and the ROIs were selected conservatively that only areas whose signals were strong in attenuation or dark-field were classified as nodule or parenchyma. Therefore, the detectability index measured here will be slightly higher than the true value since the biological variability was suppressed. After selected, the ROIs are median-filtered with a radius of 2 pixels before the $d^{\prime}$ calculation. The mean value and standard deviation are then measured for the nodule and normal parenchyma to calculate the ${d}^\prime$.

### Histology

Histology analysis was conducted to study the autolysis happened in mice’s lung after death, whose detail will be given in dedicated subsection below. Another four mice (as mentioned above) were euthanized, and their lung tissues were dissected at 2, 4, 6, and 8 h postmortem. These lung tissues were fixed in 10% formalin solution (Damao, Tianjin, China). Paraffin sections were prepared with a thickness of 4 μm and then subjected to hematoxylin and eosin staining (Hushi, Shanghai, China). Then, the slices were dehydrated and scanned under microscope.

### Statistical analysis

Statistical analysis was conducted using GraphPad Prism (Version 9.0.0, GraphPad Software Inc., USA). To prove dark-field’s superiority to attenuation in nodule detection, whose detail will be given in dedicated subsection below, a paired *t*-test [45] was performed on the detectability index of dark-field and attenuation modalities measured on different samples. If the detectability index of dark-field modality is significantly higher than that of attenuation modality, then it can be proved that dark-field modality is better than attenuation in nodule detection.

Statistical analysis was also conducted to disprove the influence of autolysis, whose detail will be given in dedicated subsection below. An ROI was selected at the same position in the first and last dark-field projection signal from one experiment, and the dark-field signal of the pixels in these two projections were selected. The null hypothesis is that the signals in the last projection were lower than those in the first projection, which means our experiments were influenced by autolysis. One-sided paired *t*-test [45] was applied to these two groups of signals to test whether the null hypothesis is valid. If not, the impact of autolysis can be excluded.

## Results

### Dark-field and attenuation CT

**Fig. 2:**
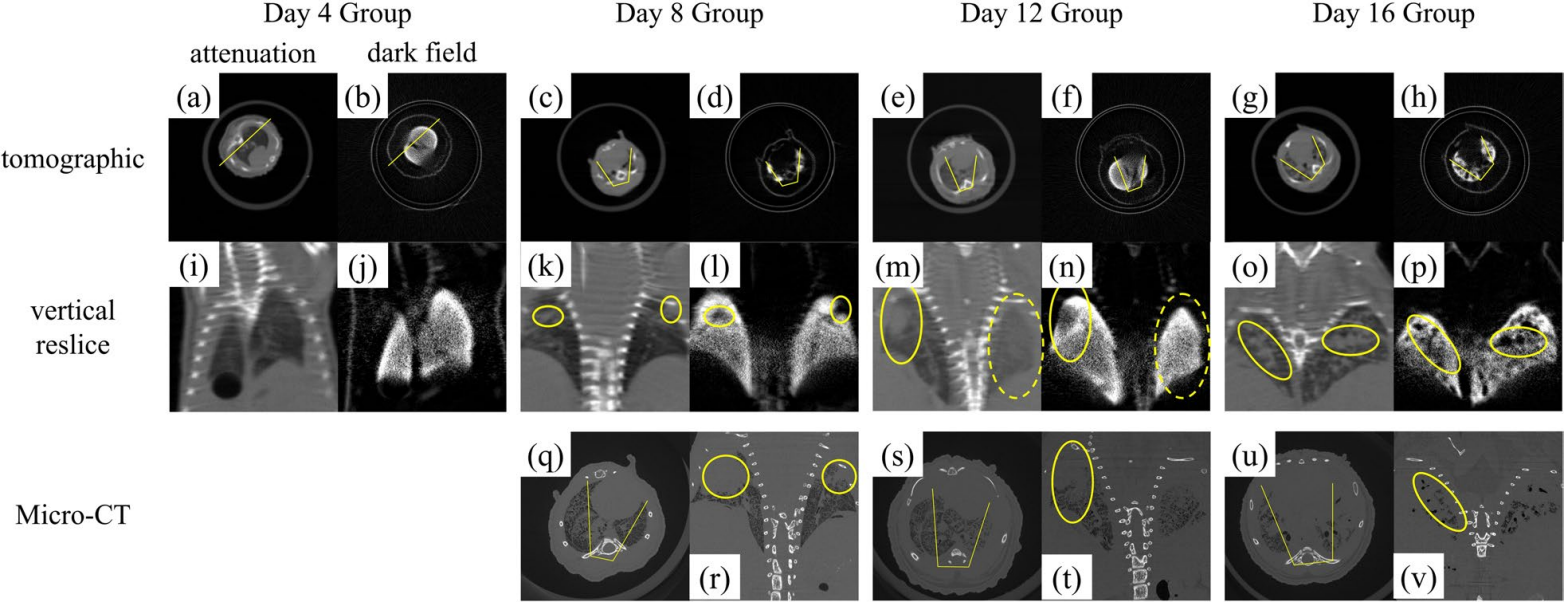
Comparison of the standard attenuation and dark-field modalities and micro-computed tomography (micro-CT) for one of the mice in each group. Images from **a** to **h** illustrate the tomographic images, and images from **i** to **p** illustrate the vertical reslices for the standard attenuation and dark-field modalities. The greyscale window was set between (0, 1.5) for the standard attenuation modality and between (0, 8) for the dark-field modality. The vertical reslices were generated from the tomographic images according to the section profile indicated by the yellow line. The yellow circles indicate the lung nodule on the vertical reslices. Images from **q** to **v** illustrate the tomographic and vertical reslices of the micro-CT images acquired on days 8, 12, and 16

Images acquired using the dark-field CT and micro-CT for one of the mice in each group are compared in Fig. 2. The CT results contain the typical tomographic slices of the standard attenuation and dark-field modalities and the vertical reslices according to the section profile shown in the tomographic slices. The micro-CT slices were used to account for the lack of spatial resolution in the standard attenuation modality. The micro-CT was manually matched with the dark-field CT. Note that the two samples in each group share the same results, so we selected one sample from each group and presented it here.

No lung nodule formation was noted on day 4 on any of the images. However, the number of nodules increased from day 8 to day 16, indicating disease progression. Generally, a signal increase in the attenuation modality corresponded with a signal decrease in the dark-field modality, indicating the nodules (yellow circle, Fig. 2), except for day 12, whereby an increase in the attenuation signal did not correspond with a decrease in the dark-field signal (dashed-line circle, Fig. 2).

The tumor characteristics in different stages of cancer progression were also studied. The number of nodules were counted according to both attenuation and dark-field data. The size of nodules was measured as per the Fleischner 2017 guideline [46]. In this guideline, the biggest section of a nodule is selected among transverse, coronal, and sagittal reconstruction data, and the size of the nodule is represented by the average of the long and short axis diameters of the nodule’s biggest section. The number and average size of the nodules in different groups are listed in Table 1. It can be observed that the number of nodules increases with the progression of cancer, while the size of nodules only slightly increases.

**Table 1:** Number and size of nodules in different groups

Group	Number of nodules	Size of nodules (mm)
Day 8	8	1.461 ± 0.200
Day 12	10	1.547 ± 0.252
Day 16	17	1.644 ± 0.491

The size of nodules is represented as the mean ± standard deviation

### Quantitative analysis

**Fig. 3:**
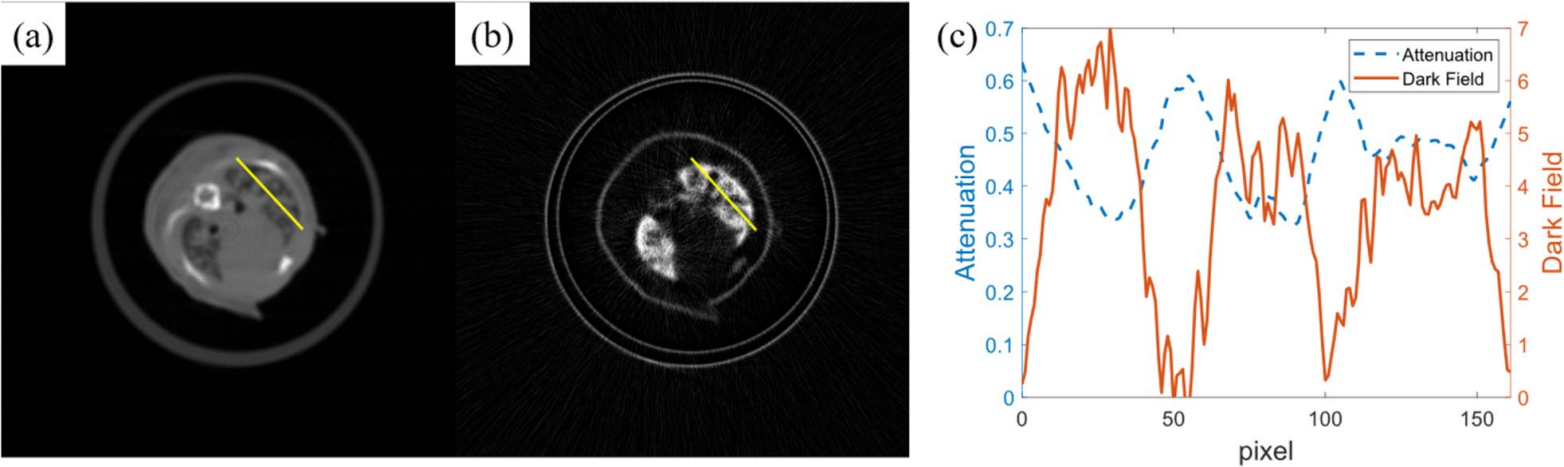
A typical tomographic slice of attenuation (**a**) and dark-field modality (**b**) obtained from one of the samples in the day 16 group. **c** The attenuation and dark-field profile for the pixels indicated by the yellow line in **a** and **b**

Generally, nodules have a low dark-field signal and high attenuation signal, and vice versa for normal pulmonary parenchyma. However, as shown by the line profile in Fig. 3, the contrast between the nodule and normal tissue was higher in the dark-field modality than that in the standard attenuation modality.

**Fig. 4:**
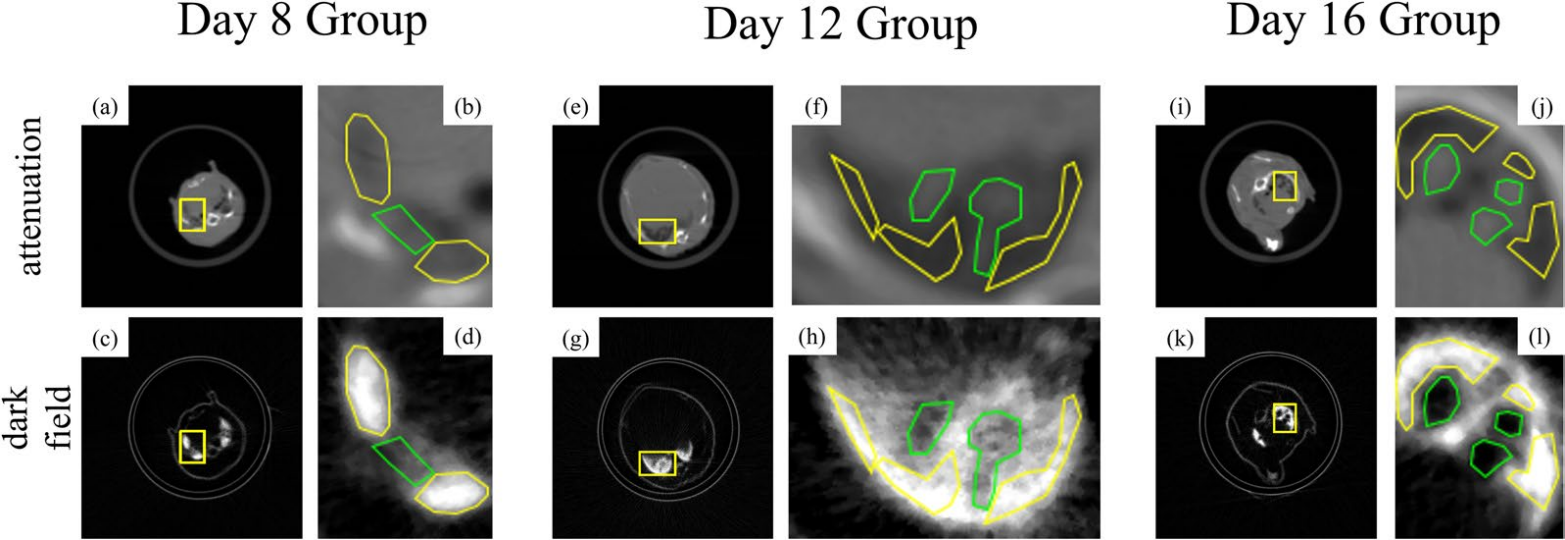
Typical slices of attenuation and dark-field modality of the day 8 (**a**-**d**), day 12 (**e**–**h**), and day 16 (**i**–**l**) groups. For each group, a yellow rectangle was drawn on both images at the same place to mark the ROI, and the areas were zoomed and then median-filtered with a radius of 2 pixels. The green ROI indicates the nodules, while the yellow region of interest indicates the normal lung parenchyma

To measure the detectability index, ROIs of normal parenchyma and nodules were selected, as shown in Fig. 4, and the $d^\prime$ was calculated according to Eq. 2, as shown in Table 2. Overall, the dark-field modality showed a higher detectability index than the standard attenuation modality, especially in day 8.

**Table 2:** Detectability index of attenuation and dark-field modality of lung nodules

Group	Imaging modality	Nodules		Normal pulmonary parenchyma		Detectability index
		Mean	SD	Mean	SD	
Day 8	Attenuation	0.517	0.039	0.500	0.039	0.436
	Dark-field	2.421	0.681	6.512	1.271	4.012
Day 12	Attenuation	0.434	0.044	0.311	0.028	3.335
	Dark-field	4.089	0.925	7.222	0.951	3.340
Day 16	Attenuation	0.558	0.030	0.368	0.061	3.953
	Dark-field	0.691	0.617	7.074	1.132	7.002

*SD* Standard deviation

Following the same calculation method, the detectability indices of all the mice with observable nodules are listed in Table 3, including groups from day 8 to day 16. Based on these indices, statistical analysis showed significantly (*p* = 0.025) that dark-field modality outperforms attenuation in pulmonary nodule detection.

**Table 3:** Detectability index of attenuation and dark-field modality measured in all the six mice with observable nodules

Group	Detectability index	
	Attenuation	Dark-field
Day 8	0.436	4.012
	3.728	4.626
Day 12	3.335	3.340
	6.557	7.529
Day 16	3.953	7.002
	4.172	6.524

### Autolysis analysis

**Fig. 5:**
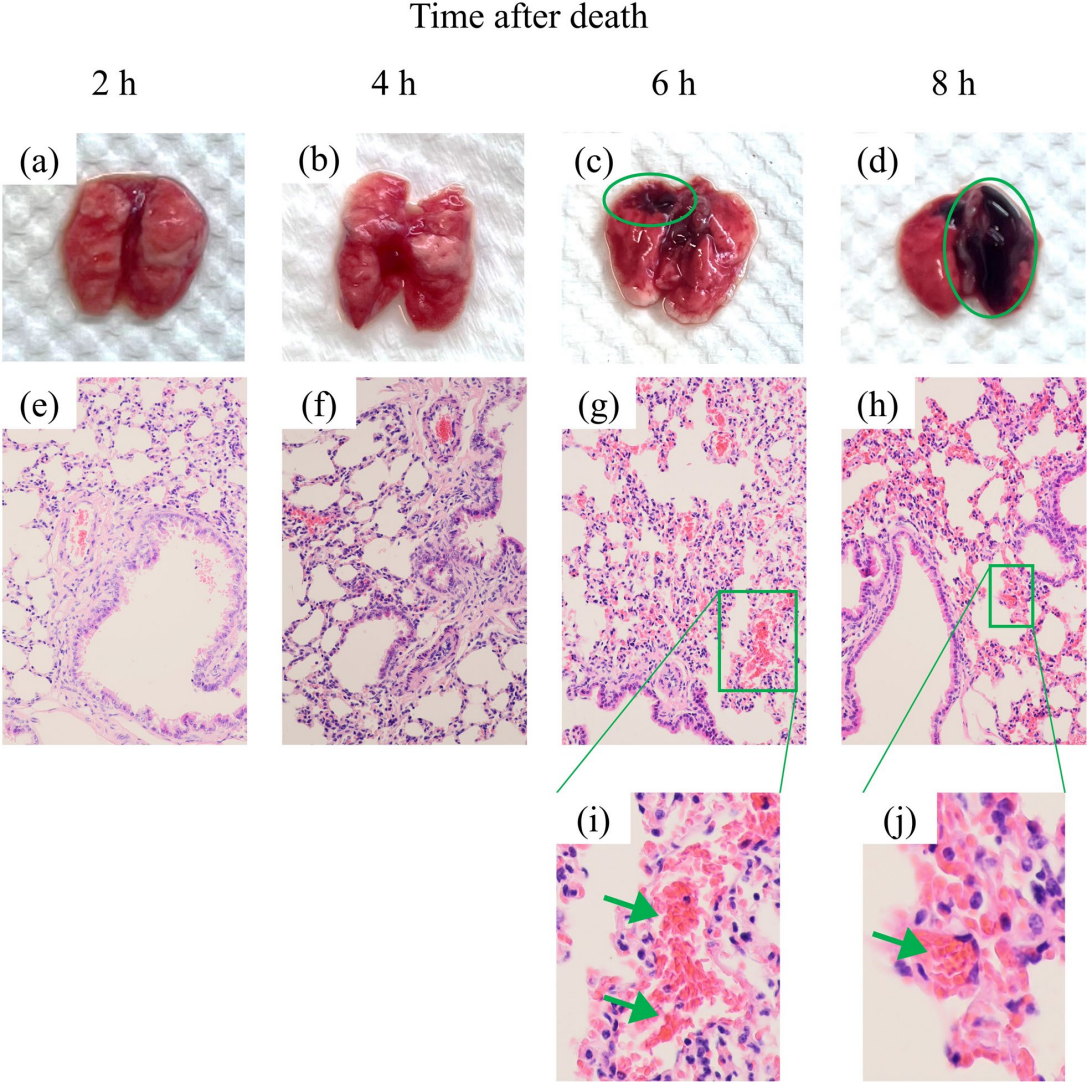
The pulmonary autopsy (**a**–**d**) and pathological histology (**e**–**h**) of mice at 2, 4, 6, and 8 h after death. Pulmonary autolysis can be observed after 6 h. Dark spots (green ellipses) can be observed in autolysis results (**c**, **d**). Correspondingly, erythrocyte leakage can be observed in the histology results (**g**, **h**), which are zoomed in and marked with a green arrow in **i** and **h**

The mice underwent pulmonary autolysis after euthanized [47]. To study the timing of autolysis, we evaluated the onset of pulmonary autolysis in healthy mice through histology. The results of this analysis are shown in Fig. 5. Dark spots are shown on autopsied lung tissue since 6 h postmortem, and it can be observed that autolysis occurs at the location of the dark spots under microscope, which can be visualized on histological slices as erythrocyte leakage, and pyknosis of nuclei [48]. The erythrocyte leakage is indicated with a green arrow in Fig. 5i and h, and the pyknosis of nuclei can be determined by comparing the degree of nuclear staining at different time postmortem in Fig. 5e–h. Based on this analysis, we found that lung autolysis can be observed at 6 h after the death of the mice.

**Fig. 6:**
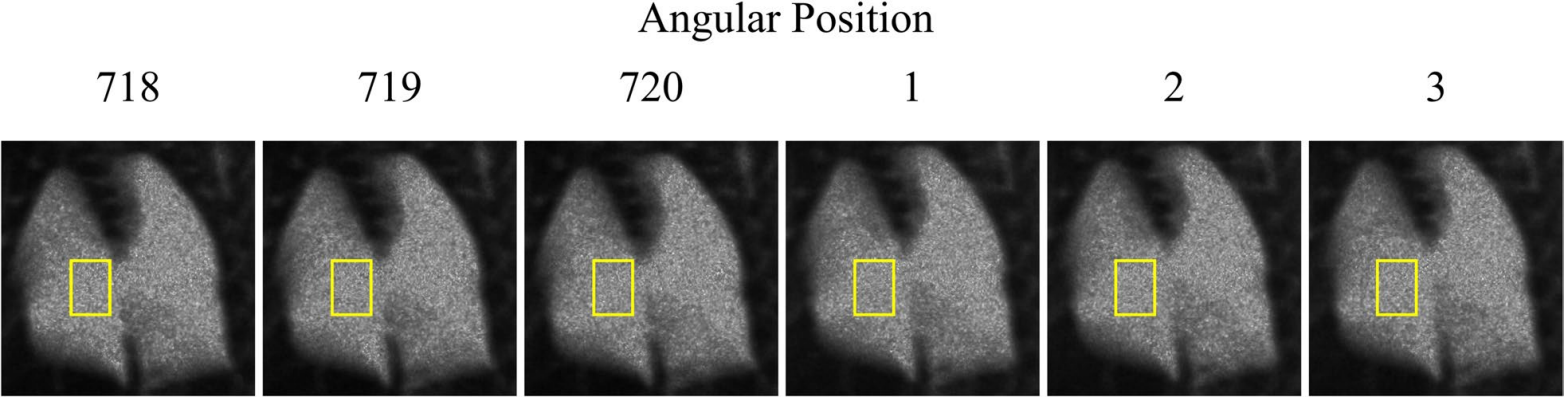
The 718^th^ to 720^th^ and 1^st^ to 3^rd^ projection of one of the mice in the day 4 group. The yellow rectangle indicates the region of interest used to calculate the mean signal and standard deviation of the dark-field signal. The greyscale window was set between (0, 6.28)

Considering the 8-h-long scan time of our CT, lung autolysis occurred during the experiment. As a result, the destruction of the alveoli structure during the experiment could have altered the dark-field signal and led to artifacts. To confirm whether our experiment was affected by autolysis, we compared the dark-field signal between the first and last several projections on one of the mice in day 4 group (Fig. 6). These projections were adjacent in angle but had a longer interval time. However, as shown in Table 4, the average dark-field signal fluctuated according to the angular position, which contradicted the possibility of autolysis where the dark-field signal would decrease in the experiment.

**Table 4:** Mean and standard deviation of the dark-field signal of the region of interest drawn in Fig. 6 for the adjacent angular positions

Angular position	Mean	Standard deviation
718	3.356	0.504
719	3.376	0.486
720	3.343	0.466
1	3.312	0.462
2	3.371	0.477
3	3.306	0.480

Statistical analysis was also conducted to further disprove the impact of autolysis. The pixels in ROIs of the first and last projections in Fig. 6 were selected, and one-sided paired *t*-test was applied to the dark-field signal of these two ROIs as mentioned above. *T*-test indicated significantly (*p* = 0.007) that the dark-field signals in the last projection were not lower than those in the first projection, which showed that the dark-field signal in the same position did not decrease during the experiment. Based on these findings, we concluded that lung autolysis had no significant impact on our experiment.

## Discussion

In this study, we aimed to evaluate the feasibility of using dark-field CT to detect nodules at different stage of development using a lung cancer mouse model. Overall, our findings indicate that the dark-field modality complemented with the attenuation modality, and they revealed the micro-structural difference between nodules and normal pulmonary parenchyma. However, the dark-field modality exhibited a larger contrast between the pulmonary nodules and normal parenchyma. These findings were also confirmed by the quantitative analysis, which also showed that the lung nodule on the dark-field modality had a higher detectability index than the conventional attenuation modality after median filtering. This advantage was more pronounced in the day 8 group. These findings indicate that dark-field is better at detecting lung nodules at an early stage than the standard attenuation modality when the nodules are obvious in dark-field modality but not in attenuation modality.

We also conducted micro-CT to achieve high-resolution imaging for comparison and cross-validation with our dark-field CT. The acquisition time of our dark-field CT is longer than that of the micro-CT mainly due to the delay caused by mechanical movement and incompletely optimized data readout from the detector. However, micro-CT has several disadvantages. Although micro-CT has a better spatial resolution, it requires denser pixels for spatial resolution, thus producing a higher radiation dose at the same statistical noise level [44]. Furthermore, image registration is hard to perform between micro-CT and dark-field modality due to different experimental setups, so it can only provide an approximate comparison, while in dark-field CT, the dark-field and attenuation modalities are retrieved from the same raw data captured by the detector, so these two modalities have an intrinsically perfect registration.

As for the above-mentioned abnormality of the day 12 group, the ground-glass opacity of the day 12 group in attenuation modality in Fig. 2e, m can be attributed to inflammation or blood vessel bursting. However, we could not compare the imaging findings with pathological data since pulmonary autolysis occurred after the death of the mice. Theoretically, ground-glass opacity represents the reduction of alveoli intervals, which will also lead to the reduction of dark-field signal. However, the signal of the two lungs has no obvious difference, while ground-glass opacity only exists in one of the two lungs. We attributed this finding to the reconstruction noise or low dark-field sensitivity of the current system.

Our current study has some limitations that have to be acknowledged. The limited hardware available and the large focal spot used in our study reduced the quality of the images. Several ring and radial artifacts were observed on tomographic images, which could be attributed to numerous factors, including non-uniform detector response, anisotropic scattering of the lung, phase stepping jitter, and focus drift. As shown in our quantitative study, since the dark-field signal is related to the visibility of the phase stepping curve, it is more sensitive to noise in the phase stepping than the attenuation modality. However, the use of median filtering in our study reduced the high-frequency noise in the reconstruction images, leading to an increase in the detectability index of dark-field modality. Besides, although each group consisted of two randomly selected mice, the potential influence of inherent confounding variables in mice is not completely eliminated. Furthermore, due to the lack of histological analysis, the impact on diagnosis and treatment allocation by the improvement in detectability index of dark-field CT is so far unclear, which should be studied in the future work.

In our study, we had no strict control group, as all mice were inoculated with cancer. However, consistent with previous studies, no nodules were observed on the images on day 4 since the B16 cell lines started to produce cancer after 7 days. Therefore, no signal inhomogeneity was noted in the day 4 group.

Overall, we successfully used dark-field CT to monitor tumor progression in mice. Our findings suggest that dark-field CT might provide a promising, imaging modality to diagnose lung disease. This imaging modality could potentially improve diagnosis of lung diseases especially at early stage. Considering the latest development of dark-field CT prototype for human scale with scan times of 1 s [21], our long-term goal is to extend the lung disease diagnosis with dark-field CT to human scale.

## Data Availability

The datasets used and analyzed during the current study are available from the corresponding author on reasonable request.

## Abbreviations

CT: Computed tomography
ROI: Region of interest
